# Chemoresistance in H-Ferritin Silenced Cells: The Role of NF-κB

**DOI:** 10.3390/ijms19102969

**Published:** 2018-09-28

**Authors:** Ilenia Aversa, Roberta Chirillo, Emanuela Chiarella, Fabiana Zolea, Maddalena Di Sanzo, Flavia Biamonte, Camillo Palmieri, Francesco Costanzo

**Affiliations:** 1 Research Center of Biochemistry and Advanced Molecular Biology, Department of Experimental and Clinical Medicine, “Magna Græcia” University of Catanzaro, Campus Salvatore Venuta-Viale Europa, 88100 Catanzaro, Italy; ilenia.aversa@unicz.it (I.A.); roberta.chirillo@unicz.it (R.C.); adry@unicz.it (M.D.S.); flavia.biamonte.fb@gmail.com (F.B.); 2 Department of Experimental and Clinical Medicine, University Magna Graecia of Catanzaro, Campus Salvatore Venuta-Viale Europa, 88100 Catanzaro, Italy; emanuelachiarella@unicz.it (E.C.); zolea.fabiana@gmail.com (F.Z.); fsc@unicz.it (F.C.); 3 Interdepartmental Center of Services (CIS), University Magna Graecia of Catanzaro, Campus Salvatore Venuta-Viale Europa, 88100 Catanzaro, Italy

**Keywords:** Ferritin Heavy Chain, NF-κB, ROS, chemoresistance

## Abstract

Nuclear Factor-κB (NF-κB) is frequently activated in tumor cells contributing to aggressive tumor growth and resistance to chemotherapy. Here we demonstrate that Ferritin Heavy Chain (FHC) protein expression inversely correlates with NF-κB activation in cancer cell lines. In fact, FHC silencing in K562 and SKOV3 cancer cell lines induced p65 nuclear accumulation, whereas FHC overexpression correlated with p65 nuclear depletion in the same cell lines. In FHC-silenced cells, the p65 nuclear accumulation was reverted by treatment with the reactive oxygen species (ROS) scavenger, indicating that NF-κB activation was an indirect effect of FHC on redox metabolism. Finally, FHC knock-down in K562 and SKOV3 cancer cell lines resulted in an improved cell viability following doxorubicin or cisplatin treatment, being counteracted by the transient expression of inhibitory of NF-κB, IκBα. Our results provide an additional layer of information on the complex interplay of FHC with cellular metabolism, and highlight a novel scenario of NF-κB-mediated chemoresistance triggered by the downregulation of FHC with potential therapeutic implications.

## 1. Introduction

Resistance to chemotherapy critically impairs the efficacy and outcome of cancer drugs. In the last years, much progress has been made to delineate the mechanisms of cellular resistance, which also includes the modulation of regulatory protein expression [1]. The nuclear factor-κB (NF-κB) family of transcription factors has been identified as a key player in resistance mechanisms [2]. Indeed, many evidence over the last few years indicate that most chemotherapeutic agents activate NF-κB in vitro and in vivo mechanisms (reviewed in [2]). Moreover, induction of chemoresistance is mediated through several genes regulated by NF-κB [3] and inhibition of this transcription factor increases sensitivity of cancer cells to the apoptotic action of chemotherapeutic agents [4].

NF-κB consists in a family of structurally related monomers RelA (p65), RelB, c-Rel, p50, and p52, which assemble into a variety of functional homo and heterodimers with distinct physiological functions and gene targets [5,6]. Complex regulatory networks exist to ensure that NF-κB is activated only by appropriate stimuli [7]. In the canonical pathway, NF-κB typically resides in the cytoplasm in an apparently inhibited state, bound to the α-isoform of Inhibitor of NF-κB (IκBα). In response to activating stimuli, the IκBα is phosphorylated and exposed to proteasomal degradation, thus releasing NF-κB proteins to their free forms. Next, NF-κB proteins are translocated to the nucleus where, with the help of co-regulators, they will orchestrate the cascade of signaling responses to the external stimuli.

Ferritin is a ubiquitous iron storage protein present in mammalian serum and tissues [8]. In eukaryotic cells ferritin is distributed in cytoplasm, nucleus, and mitochondria [9]. The nanocage of cytoplasmic ferritin is composed by 24 subunits of light (FTL) and heavy (FHC, Ferritin Heavy Chain) types [9], coded by different genes whose expression might be finely tuned at transcriptional [10,11,12] and post-transcriptional levels [13,14]. FTL and FHC, despite showing extensive sequence homology, perform different roles in iron metabolism. FHC holds a ferroxidase activity and is mainly implicated in iron uptake and release, while FTL is devoted to the long-term iron storage [9]. As free iron contributes to the generation of reactive oxygen species (ROS) through Fenton’s reaction, the enzymatic activity of FHC makes this protein a key component of redox metabolism [15].

Several links have been so far identified among iron/redox metabolism, NF-κB and ferritin. Among others, it has been shown that: (i) The complex cascade of phosphorylation and de-phosphorylation events leading to NF-κB activation is intimately linked to the redox status of the cell [16]; (ii) the direct addiction of iron to culture medium of Kupffer cells activates NF-κB pathway [17]; (iii) iron chelators are able to inhibit NF-κB activation induced by endotoxin in hepatic macrophages [18]; and (iv) iron overload modulates NF-κB activity in endometrial stromal cells [19].

In previous papers, we demonstrated that knockdown of FHC in in two transformed cell line, K562 erythroleukemia cells and SKOV3 ovarian carcinoma cells, was accompanied by the altered expression of a number of genes [20] and of a repertoire of oncomiRNAs [21,22], resulting in altered proliferation, apoptosis and stem cell expansion [22,23,24]. Interestingly, ingenuity pathway analysis (IPA) of some of these FHC-dependent genes [20], as well as IPA networks of the genes targeted by miR-675 [25], one of the putative FHC-dependent miRNAs, highlighted NF-κB as central hub of the predicted metabolic routes. In this study, we explored the relationship between FHC and NF-κB in K562 and SKOV3 ovarian carcinoma cells, and its impact on chemoresistance to antineoplastic agents.

## 2. Results and Discussion

### 2.1. Ferritin Heavy Chain (FHC) Expression Inversely Correlates with P65 Nuclear Accumulation 

**Figure 1:**
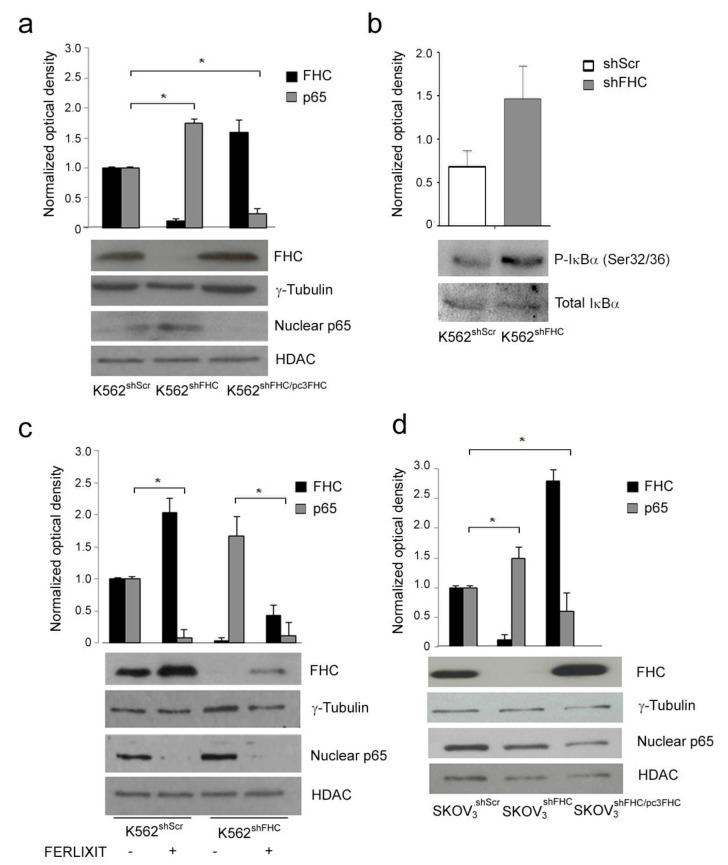
Western Blot analysis of Ferritin Heavy Chain (FHC) and nuclear p65 expression in K562 and SKOV3 cell lines. (**a**) Western Blot analysis of FHC and nuclear p65 expression in K562^shScr^, K562^shFHC^ and K562^shFHC/pcFHC^ cells. (**b**) Western Blot analysis of phospho-IκBα S32/36 in K562^shScr^ and K562s^hFHC^ cells. (**c**) Western Blot analysis for FHC and nuclear p65 expression upon Ferlixit^®^ treatment of K562^shScr^ and K562^shFHC^ cells. (**d**) Western Blot analysis of FHC and nuclear p65 expression in SKOV3^shScr^, SKOV3^shFHC^ and SKOV3^shFHC/pcFHC^ cells. For all panels, FHC expression analysis was performed on 40 µg of total protein, while nuclear p65 expression was performed on 40 µg of nuclear protein extracts. γ-Tubulin, IκBα and HDAC were used as loading control for total or nuclear protein levels, respectively. Blots are representative of three independent experiments. The graph represents the mean of the optical densities (* *p* < 0.05).

In order to explore whether perturbation of intracellular amounts of FHC might alter NF-κB pathway, we first examined the correlation between the steady-state amounts of FHC and NF-κB activation in a pool of K562 cell clone stably silenced for FHC by means of a specific shRNA (K562^shFHC^) [20]. The nuclear p65 amounts were determined as a read-out of the canonical NF-κB pathway activation [5]. In FHC-silenced K562shFHC cells nuclear p65 levels almost doubled as compared to K562^shScr^ control cells (Figure 1a). Nuclear translocation of NF-κB results by IκB kinase (IKK)-mediated phosphorylation of IκBα at serines 32 and 36 followed by its proteasome-mediated degradation. The K562^shFHC^ cells showed higher levels of phospho-IκBα (Ser 32/36) than K562^shScr^ control cells (Figure 1b), further supporting the activation of canonical NF-κB pathway FHC-silenced in K562^shFHC^ cells.

To rule out the possible off-target effects of the shRNA, and to deeply relate FHC with p65 levels, FHC was also restored in the silenced cells by transient transfection of an FHC-expression vector (K562^shFHC/pc3FHC^). Upon FHC transient over-expression, K562^shFHC/pc3FHC^ showed a strong decrease of nuclear p65 levels as compared to K562^shFHC^ (Figure 1a), indicating that p65 nuclear levels were inversely related with FHC protein levels.

To further strengthen the inverse relationship of FHC and p65, control K562^shScr^ and silenced K562^shFHC^ cells were treated with sodium ferric gluconate (Ferlixit^®^, SANOFI-AVENTIS SpA, Milano, Italy), a strong inducer of Ferritin mRNA translation [8,26]. The Ferlixit treatment (0.5 µM for 24 h) induced a consistent increase of FHC protein amounts in control and silenced cells that, in turn, was accompanied by down-regulation of p65 nuclear accumulation (Figure 1c). These results demonstrated an inverse correlation of FHC and NF-κB activity, being the nuclear p65 protein increased in the FHC-silenced cells and strongly reduced upon transfection of ferritin expression vector.

To evaluate whether the inverse correlation of FHC and NF-κB activity was restricted to K562 erythroleukemia cells, we also analyzed nuclear p65 in a pool of stably FHC-silenced SKOV3 human ovarian adenocarcinoma cells (SKOV3^shFHC^). Similarly to the results observed on K562^shFHC^ cells, the steady-state amount of nuclear p65 in SKOV3^shFHC^ was about twice, as compared to control SKOV3^shRNA^ cells, and was strongly down-regulated upon FHC reconstitution (SKOV3^shFHC/pc3FHC^). (Figure 1d), indicating that this phenomenon may be shared by different cancer cell lines.

An intriguing ability of FHC is to function as an inhibitor [23] or as an activator [22] of given metabolic routes. FHC manifest inhibitory activity, for instance, in the case of DAXX-mediated apoptosis [27], while it is a strong activator of the molecular events leading to epithelial to mesenchymal transition [22]. Taken all together, our results in K562 and SKOV3 cells indicated that the NF-κB pathway might be added to those in which FHC function as negative regulatory hub.

### 2.2. FHC Modulates NF-κB Activation through Reactive Oxygen Species (ROS) Increase

**Figure 2:**
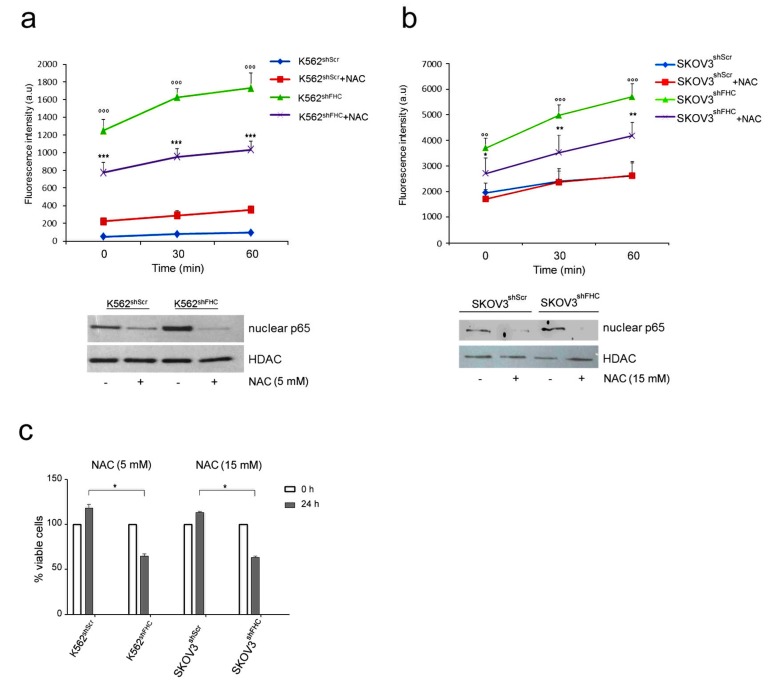
Reactive oxygen species (ROS) levels and p65 expression in K562 cells after treatment with NAC. (**a**) Upper panel: ROS analysis of K562^shScr^ and K562^shFHC^ cells. Cells (10^6^) were treated with *N*-Acetyl-l-cysteine (NAC, 5 mM) for 1 h, incubated for 15 min with 20 µM of 2′-7′-dichlorofluorescein diacetate (DCF) and washed with Hanks’ Balanced Salt solution (HBSS). Fluorescence was measured using the Victor3 Multilabel Counter at 0, 30 and 60 min. The assay was performed in triplicate and data are represented as mean ± Standard Deviation (SD); (*** *p* < 0.001, compared with K562^shScr^, °°° *p* < 0.001, compared to K562^shFHC^); lower panel: Western Blot analysis for p65 expression, HDAC protein level was used as loading control. (**b**) Upper panel: ROS analysis of SKOV3^shScr^ and SKOV3^shFHC^ cells. Cells (10^6^) were treated with NAC (15 mM) for 15 min, incubated for 15 min with 20 µM of 2′-7′-DCF and washed with HBSS solution. Fluorescence was measured using the Victor3 Multilabel Counter at T0, T30 and T60 min. The assay was performed in triplicate and data are represented as mean ± SD; (* *p* < 0.05, compared with SKOV3^shScr^, ** *p* < 0.01, compared with SKOV3^shScr^, °° *p* < 0.01, compared to SKOV3^shFHC^, °°° *p* < 0.001, compared to SKOV3^shFHC^); lower panel: Western Blot analysis for p65 expression, HDAC was used as loading control. (**c**) Cell viability of K562 and SKOV3 cells treated with NAC for 24 h. K562 cells were treated at final concentration of 5 mM; SKOV3 cells were treated at final concentration of 15 mM. Cells viability was assayed by Trypan blue exclusion in triplicate and data are represented as mean ± SD; (* *p* < 0.05, compared with K562^shScr^ and SKOV3^shScr^).

An impaired expression of FHC is responsible of the increase of the labile iron free pool and generation of reactive oxygen species (ROS) [9]. In previous works, we demonstrated that FHC knock-down is accompanied, among other phenomena, by increased ROS production in different cell lines [25]. Since ROS have been reported to affect the activity of NF-κB [28], we asked whether the increased NF-κB activity in FHC-silenced cells was due to an indirect effect mediated by ROS. To this end, we analyzed p65 NF-κB nuclear levels in K562^shFHC^ and in SKOV3^shFHC^ cells after treatment with the ROS scavenger *N*-Acetyl-L-cysteine (NAC). FHC knock-down induced a dramatic increase of ROS levels in K562^shFHC^ as compared to K562^shScr^ control cells (Figure 2a), and about triples their content in SKOV3^shFHC^ cells, as compared to control SKOV3^shScr^ cells (Figure 2b); in both the FHC-silenced cell lines NAC treatment roughly halves the content of ROS, without bringing it back to the basal levels. While the restoration is only partial, however, the decrease of ROS content is sufficient to drastically reduce, in both cell lines, the amount of nuclear p65 (Figure 2a,b low panels). This result indicates that alteration of redox metabolism induced by FHC-silencing is the molecular link between FHC and NF-κB activity. Interestingly, NAC treatment also affected cell viability of both K562^shFHC^ and SKOV3^shFHC^ cells, which were significantly decreased as compared to control cells (Figure 2c), thus supporting a pro-survival role of the FHC-ROS-NF-κB axis in both cell lines.

Many mechanisms have been proposed to explain the influence of ROS on NF-κB signalling pathway [28], including the direct regulation of NF-κB DNA binding activity [3,7] or the regulation of upstream NF-κB activating pathways [16]. In addition, the molecular mechanisms that lead ROS to interfere with NF-κB pathway are reported to be cell-type specific [28]; it has been shown that ROS can activate NF-κB signalling in oral squamous carcinoma [29] and in HepG2 cells [30], while they act as inhibitors in A549 [31] and in ECV304 cells [32]. 

We have detected this phenomenon in hematological- and in epithelial-derived cell lines; since FHC and NF-κB are ubiquitously expressed we suggest that, with few exceptions, an imbalance in the intracellular content of the ferritin heavy subunit might induce NF-κB and its downstream pathway in a multiplicity of cell types.

### 2.3. FHC-Induced NF-κB Activation Correlates with Resistance to Chemotherapies in K562 and SKOV3 Cells

In the plethora of biological effects resulting from NF-κB activation is also included the onset of chemoresistance; platinum-based anticancer drugs, antracyclines, and taxanes are all able to induce activation of NF-κB, which in turn activates transcription, among others, of anti-apoptotic genes [32,33,34]. Consequently, in the vast majority of cases NF-κB activity is inversely related with drug sensitivity [2].

**Figure 3:**
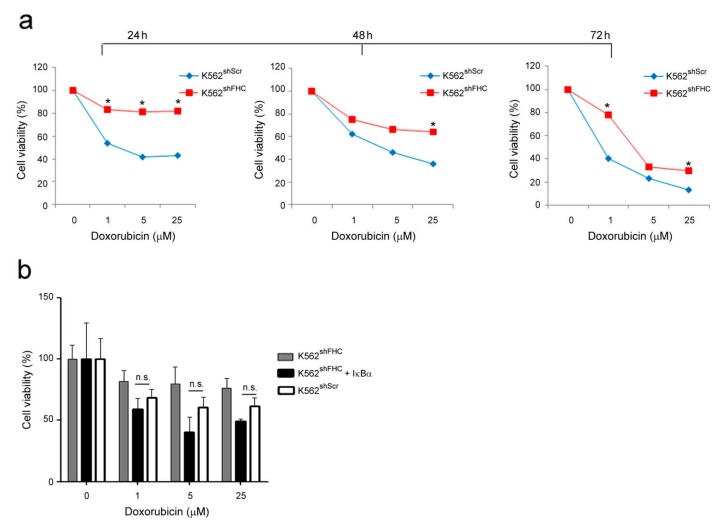
Cell viability of K562 cells treated with Doxorubicin. (**a**) Cell viability was assessed using the 3-[4,5-Dimethylthiaoly]-2,5-diphenyltetrazolium bromide (MTT) method on K562^shScr^ and K562^shFHC^ cells treated with Doxorubicin at the indicated doses. Final results represent mean ± SD of three independent experiments each performed in triplicate (* *p* < 0.05; n.s., not significant, *p* ≥ 0.05). (**b**). Cell proliferation was assessed using the MTT method on K562^shScr^, K562^shFHC/pRc/CMV^ and K562^shFHC/Rc/CMV-HA-IκBα^ cells treated with Doxorubicin at the indicated doses and transfected with IκBα. Final results represent mean ± SD of three independent experiments each performed in triplicate; n.s., statistically not significant, *p* ≥ 0.05).

We wondered whether the activation of NF-κB following FHC-silencing might confer chemoresistance to the K562 and SKOV3 cells. To this end, we performed 3-[4,5-Dimethylthiaoly]-2,5-diphenyltetrazolium bromide (MTT) assay to assess cell viability of K562 cells treated with increasing doses of Doxorubicin. Cell viability of FHC-silenced K562^shFHC^ cells was significantly improved as compared to control K562^shScr^ cells, being about twice at all drug concentrations utilized after 72 h (Figure 3a). The increased chemoresistance of K562 FHC-silenced cells was largely mediated by the NF-κB activity; indeed, the transient transfection of the NF-κB inhibitor IκBα in these cells (K562^shFHC/Rc/CMV-HA-IκBα^) almost completely abolishes the acquired chemoresistance, bringing the cell viability to the levels of control cells at 24 h of treatment (Figure 3b). 

**Figure 4:**
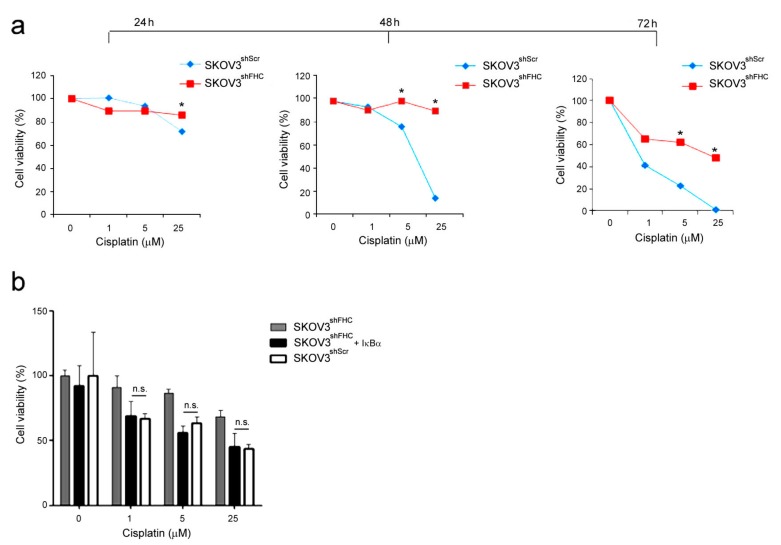
Cell viability of SKOV3 cells treated with Cisplatin. (**a**) Cell proliferation was assessed using the MTT method on SKOV3^shScr^ and SKOV3^shFHC^ cells treated with Cisplatin at the indicated doses. Final results represent mean ± SD of three independent experiments each performed in triplicate (* *p* < 0.05; n.s., not significant, *p* ≥ 0.05). (**b**) Cell proliferation was assessed using the MTT method on SKOV3^shFHC/pRc/CMV^ and SKOV3^shFHC/Rc/CMV-HA-IκBα^ cells treated with Cisplatin at the indicated doses and transfected with IκBα. Final results represent mean ± SD of three independent experiments each performed in triplicate (n.s., not significant, *p* ≥ 0.05).

Parallel experiments were performed with SKOV3 ovarian cancer cells, using Cisplatin, a specific platinum-based antineoplastic drug for ovarian carcinoma. Cell viability of FHC-silenced SKOV3^shFHC^ cells was significantly higher than control SKOV3^shScr^ cells at high doses (five and 25 µM), with the most consistent difference in cell viability recordable at 48 and 72 h of treatment (Figure 4a). Transient transfection of IκBα expression vector significantly sensitizes SKOV3^shFHC^ cells to drug at 48 h treatment (Figure 4a), thus suggesting again a central role of activated NF-κB pathway in this phenomenon. 

**Figure 5:**
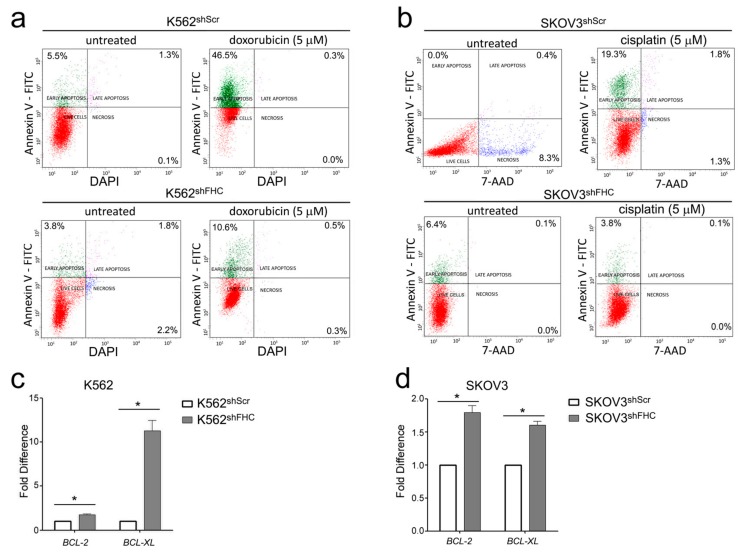
(**a**) Representative plots of Annexin V/DAPI apoptosis assays in K562^shScr^ and K562^shFHC^. (**b**) Representative plots of Annexin V/7-AAD apoptosis assays in SKOV3^shScr^ and SKOV3^shFHC^. Fluorescence-activated cell sorting (FACS) analysis was performed on three independent biological replicates. (**c**,**d**) Real-time PCR analysis of *BCL-2* and *BCL-xL* mRNAs expression were performed on total RNA extracted from K562^shScr^, K562^shFHC^, SKOV3^shScr^, and SKOV3^shFHC^ cells. Final results represent mean ± SD of three independent experiments. Statistical significance was evaluated by student t-test (* *p* < 0.05).

Doxorubicin treated K562^shScr^ cells showed an increased number of Annexin V-positive and vital dye-negative cells as compared to untreated K562^shScr^ cells, a flow cytometric profile consistent with early apoptosis (Figure 5a). The fraction of apoptotic cells were strongly reduced in doxorubicin treated FHC-silenced K562^shFHC^ cells (Figure 5a). Similar results were observed in cisplatin-treated SKOV3 cells (Figure 5b).

Then, we measured the expression of *BCL-2* and *BCL-xL*, two classical anti-apoptotic NF-κB-dependent genes [35] upon drug treatment, which resulted to be both upregulated in FHC-silenced K562 and SKOV3 cells, thus possibly accounting for the reduced apoptosis of FHC-silenced cells (Figure 5c,d).

The existence of a direct correlation between FHC and NF-κB is known since 1995, when Kwak et al. showed that FHC is transcriptionally regulated by NF-κB [36]. The set of our results in hematological and epithelial-derived cancer cell lines indicated that NF-κB might be also positioned downstream FHC, as well as upstream, thus suggesting the existence of a feedback loop between these two proteins. A similar loop also exists between FHC and p53, as recently reviewed by Min Pang and Connor [37]. p53, indeed, modulates transcription of FHC which, in turn, might bind and modulate the activity of the tumor suppressor. FHC plays a complex role in apoptosis; on the one hand its antioxidant functions confer anti-apoptotic activity to the molecule, on the other the activation of p53 pathway configures FHC as pro-apoptotic protein. Consequently, it can be assumed that FHC might either drive or counteract apoptotic pathway in function of the cell type as well as of the cell context; our findings in K562 and SKOV3 cells suggested a role of FHC as pro-apoptotic protein trough NF-κB pathway activation, adding an additional layer of information on the complex interplay of FHC with cellular metabolism. 

## 3. Conclusions

In our previous works we demonstrated that FHC knock-down is accompanied by increased ROS production [25], which drives the transition toward a mesenchymal phenotype in MCF-7 and H460 carcinoma cells [23], or determines a severe protein misfolding in K562 erythroleukemia cells [15]. In this study, we provide an additional layer of information on the complex interplay of FHC, and thus iron metabolism, in cancer biology [38], showing that the downregulation of FHC correlates with an increased chemoresistance of K562 and SKOV3 cells. 

To our knowledge only one study has shown the correlation between FHC expression and chemoresistance in patients. In particular, Y Zheng et al [39] reported that FHC expression inversely correlates with resistance to SN38 (the active metabolite of the topoisomerase inhibitor Irinotecan), as part of a gene signature of drug resistance which would predict patient survival in colorectal cancer. Collectively, our findings support the existence of a FHC-ROS-NF-κB axis, which would account for the acquired chemoresistant phenotype in K562 and SKOV3 cell line models, encourages a deeper investigation of the correlation between FHC expression in cancer tissues and clinical outcomes, and highlights the potentiality of FHC in cancer therapy.

## 4. Materials and Methods

### 4.1. Cell Culture and Treatment

K562 cells, a human cell line established from the pleural effusion of a 53-year-old female with chronic myelogenous leukemia in terminal blast crises (ATCC number CCL-243) and SKOV3 cells, human ovarian cancer cells (ATCC, Manassas, VA, USA) were cultured in RPMI 1640 medium supplemented with 10% fetal bovine serum and antibiotics (Sigma Aldrich, St. Louis, MI, USA) at 37 °C in an atmosphere of humidified air containing 5% CO_2_. 

Lentiviral preparations and transductions were performed as previously described in Reference [20] using a shRNA as control or a shFHC that targets the 196–210 region of the FHC mRNA. All the experiments were performed using a puromycin-selected pool of clones (1 µg/mL) (Sigma Aldrich). 

K562 cells were transfected using the Nucleofector system from Amaxa (Lonza, Basel, Switzerland) according to the manufacturer’s optimized protocol. In particular, to evaluate the role of NF-κB in inducing chemoresistance and EMT-like features, we over-expressed the NF-κB inhibitor IκBα. To this end we used a homemade pRc/CMV-HA-IκBα plasmid and its scrambled control kindly provided by Ileana Quinto (Magna Graecia University of Catanzaro, Italy). To study the role of FHC amounts in controlling NF-κB activation instead we transfected K562^shFHC^ cells with the expression vector containing the full length of human FHC cDNA (pc3FHC) for 48 h. In brief, 1 × 10^6^ cells were suspended in 100 μL of pre-warmed Nucleofector Solution Kit V (Amaza, Basel, Switzerland), and mixed with 3 µg of the expression vectors or with 3 µg of scrambled controls as previously described by Chiarella et al [40]. Then, DNA-cell suspensions were transferred to Amaxa certified cuvettes and electroporated using the Nucleofector program T-016. After nucleofection the cells were transferred immediately into pre-warmed complete RPMI medium.

Doxorubicin and Cisplatin were directly added to the K562 and SKOV3 cells culture medium respectively at a final concentration of 1, 5 and 25 µM for 24, 48 and 72 h. *N*-acetyl cysteine (NAC) was added to the K562 cells culture medium at a final concentration of 5 mM for 1 h and to the SKOV3 cells culture medium at a final concentration of 15 mM for 15 min.

### 4.2. Protein Extractions

Protein extractions were peformed on K562^shScr^, K562^shFHC^, K562^shFHC/pRc/CMV^, K562^shFHC/pRc/CMV-3HA-IκBα^, K562^shScr/pcDNA^, K562^shFHC/pcDNA^, K562^shFHC/pc3FHC^, SKOV3^shScr^, SKOV3^shFHC^, SKOV3^shFHC/pRc/CMV^, SKOV3^shFHC/pRc/CMV-3HA-IκBα^, SKOV3^shScr/pcDNA^, SKOV3^shFHC/pcDNA^, and SKOV3^shFHC/pc3FHC cells^.

Briefly, for total protein extractions, K562 and SKOV3 cells were lysed in ice-cold radioimmunoprecipitation assay (RIPA) buffer containing protease inhibitors (20 mmol/L Tris, 150 mmol/L NaCl, 1% Igepal, 0.5% sodium deoxycholate, 1 mmol/L EDTA, 0.1% sodium dodecyl sulfate (SDS), 1 mmol/L phenylmethylsulfonyl fluoride, 0.15 units/mL aprotinin, and 10 μmol/L leupeptin) (Sigma Aldrich) and after removal of the cell debris by centrifugation (12,000× *g* rmp, 30 min), the protein content was determined by the Bradford method (Bio-Rad Laboratories, Hercules, CA, USA).

p65 was detected by Western Blot analysis in nuclear fraction. 20 × 10^6^ cells were harvested by centrifugation at 1000× *g* rpm for 10 min at 4 °C and washed in PBS1X. Pellets were re-suspended in 200 µL of NP40-lysis buffer (HEPES pH 7.9 10 mM, EDTA 1 mM, KCl 60 mM, and NP40 0.2%) containing protease inhibitors. Cells were incubated for 5 min at 4 °C and then centrifuged at 3500× *g* rmp for 5 min at 4 °C. The supernatants (cytoplasmic fractions) were transferred to new tubes and the resulting nuclear pellets were washed twice in 500 µL of wash buffer (HEPES pH 7.9 10 mM, EDTA 1 mM, KCl 60 mM, protease inhibitors). Nuclear pellets were then re-suspended in 300 µL of wash buffer and in 300 µL of “Pillow Buffer” (150 µL + 150 µL saccarose 60%). Nuclei were centrifuged at 6000× *g* rmp for mina t 4 °C and re-suspended in 200 µL of resuspension Buffer (tris-HCl pH 7.8 250 mM, KCl 60 mM, protease inhibitors). Samples were subjected to three cycles of thermal shock (3 min dry ice, 3 min at 37 °C) and centrifuged at 9500× *g* rmp for 15 min at 4 °C.

### 4.3. Western Blotting Analysis

A total of 40 µg protein extract was boiled for 10 min in SDS sample buffer, separated by 12% SDS-PAGE and transferred to a nitrocellulose membrane by electroblotting. Non-specific reactivity was blocked in non-fat dry milk in Tween-PBS (5% (*w*/*v*) milk in phosphate-buffer saline (PBS) (pH 7.4) and 0.005% Tween 20) for 2 h at room temperature. The nitrocellulose membranes were incubated overnight at 4 °C with the following antibodies: (a) anti-FHC (H-53) (1:200; sc-25617, Santa Cruz Biotechnology, Dallas, TX, USA), (b) anti-p65 (C-20) (sc-372, 1:1000; Santa Cruz Biotechnology), (c) anti-HDAC (AV38530, 1:5000; Sigma-Aldrich), (d) anti-HA probe (F-7) (sc-7392, 1:1000; Santa Cruz Biotechnology), (e) anti-γ-Tubulin antibody (C-20) (1:3000; sc-7396, Santa Cruz Biotechnology), (f) anti-phospho-IκBα (Ser 32/36) (1:1000; sc-101713, Santa Cruz Biotechnology), and (g) anti-IκBα (C-21) (1:1000; sc371, Santa Cruz Biotechnology).

Membranes were incubated with horseradish peroxidase (HRP)-conjugated secondary antibodies and immunoreactive bands were visualized with the ECL Western blotting detection system (BioRad, Hercules, CA, USA). 

### 4.4. MTT Assay and Trypan Blue Exclusion Test

3-[4,5-Dimethylthiaoly]-2,5-diphenyltetrazolium bromide (MTT) (Sigma Aldrich) assay was performed to analyze cell viability of K562^shScr^, K562^shFHC^, K562^shFHC/pRc/CMV^, K562^shFHC/pRc/CMV-3HA-IκBα^, SKOV3^shScr^, SKOV3^shFHC^, SKOV3^shFHC/pRc/CMV^, and SKOV3^shFHC/pRc/CMV-3HA-IκBα^ cells untreated or treated with increasing doses of Doxorubicin or/and Cisplatin, respectively, for 24 h at indicated time points. 5 × 10^4^ cells/well were seeded into 96-well plate and let to grow for 48 h in RPMI medium. There were octuplicates for each experimental condition. Fresh MTT (Sigma Aldrich), re-suspended in PBS, was added to each well. After 4 h incubation, formazan salts were re-suspended using isopropanol. Optical density was measured at 490 nm in a spectrophotometer. Each experiment was performed in triplicate. Trypan blue exclusion test was performed to quantify the number of viable cells in samples from both the K562 and SKOV3 cell lines. The cells were seeded into 6-well plates containing RPMI growth medium at a density of 1 × 10^6^ cells/mL. The cell suspension cultures were then diluted at a ratio of 1:1 with 0.4% trypan blue solution, after which the number of viable cells were counted using a microscope.

### 4.5. ROS Detection

ROS were determined by incubating K562^shScr^, K562^shFHC^, K562^shFHC/pc3FHC^, SKOV3^shScr^, SKOV3^shFHC^, and SKOV3^shFHC/pc3FHC^ cells with the redox-sensitive probe 2’-7’-DCF (CM-H2CFDA; Molecular Probes, Eugene, OR, USA). Briefly, 1 × 10^6^ K562 and SKOV3 cells were plated in 96-well plates and incubated with Hanks balanced saline solution (HBSS), 10 mM glucose and 20 µM DCF for 15 min at 37 °C. After two cycle washes, cells were maintained in HBSS supplemented with 10 mM glucose. Fluorescence was revealed using the Victor3 Multilabel Counter (Perkin Elmer, Turku, Finland) at 485 nm and 535 nm for excitation and emission, respectively. Results were normalized on protein concentration.

### 4.6. Apoptosis Analysis

Apoptosis was evaluated by double staining with Annexin-V and vital dyes (DAPI or 7-AAD) using Alexa Fluor^®^488 Annexin V/Dead Cell Apoptosis Kit (Thermo Fisher Scientific, Waltham, MA, USA), according to the manufacturer’s instructions. After staining, cells were incubated at room temperature for 15 min in the dark. Each tube was diluted with 400 µL of Annexin Binding Buffer and then cells were analyzed by flow-cytometry using the BD LSRFortessaTMX-20 (BD Biosciences, San Jose, CA, USA) and FACSDiva 7.0 program (BD Biosciences).

### 4.7. RNA Isolation and qRT-PCR Analysis

Total RNA isolation, single-stranded complementary DNA (cDNA) generation and Relative-Quantitative RT-PCR were performed as previously reported in Aversa et al. [23]. Validated qRT-PCR primers for *BCL-2* and *BCL-xL* were from Thermo Fisher Scientific. GAPDH was used as internal normalizers for mRNA.

Absolute qRT-PCR was used to determine the expression of BCL-2 and *BCL-xL* L in K562 and SKOV3 cells. Briefly, 20 ng of cDNA was amplified in 20 μL of reaction mix containing Power SYBR Green PCR Master mix (Thermo Fisher Scientific), 20 pmol of each primer pair and nuclease-free water. The thermal profile consisted of 1 cycle at 95 °C for 10 min followed by 40 cycles at 95 °C for 15 s, and 60 °C for 1 min. The human GAPDH cDNA fragment was amplified as the internal control. Data analysis was performed using the 2^−ΔΔ*C*t^.

### 4.8. Data Analysis and Statistical Methods

All experiments were conducted at least two times, and the results were from representative experiments. Data were expressed as mean values ± SD. The Student’s t-test or analysis of variance (ANOVA) were used to compare the groups. Summary statistics are presented as the mean ± S.D. *p* ≤ 0.05 were considered statistically significant. 

## Abbreviations

FHC	Ferritin Heavy Chain
ROS	Reactive Oxygen Species
